# Developing a weighting strategy to include mobile phone numbers into an ongoing population health survey using an overlapping dual-frame design with limited benchmark information

**DOI:** 10.1186/1471-2288-14-102

**Published:** 2014-09-04

**Authors:** Margo L Barr, Raymond A Ferguson, Phil J Hughes, David G Steel

**Affiliations:** 1 Centre for Epidemiology and Evidence, NSW Ministry of Health, 73 Miller Street, North Sydney, Australia; 2 National Institute for Applied Statistics Research Australia, University of Wollongong, Wollongong, Australia

## Abstract

**Background:**

In 2012 mobile phone numbers were included into the ongoing New South Wales Population Health Survey (NSWPHS) using an overlapping dual-frame design. Previously in the NSWPHS the sample was selected using random digit dialing (RDD) of landline phone numbers. The survey was undertaken using computer assisted telephone interviewing (CATI). The weighting strategy needed to be significantly expanded to manage the differing probabilities of selection by frame, including that of children of mobile-only phone users, and to adjust for the increased chance of selection of dual-phone users. This paper describes the development of the final weighting strategy to properly combine the data from two overlapping sample frames accounting for the fact that population benchmarks for the different sampling frames were not available at the state or regional level.

**Methods:**

Estimates of the number of phone numbers for the landline and mobile phone frames used to calculate the differing probabilities of selection by frame, for New South Wales (NSW) and by stratum, were obtained by apportioning Australian estimates as none were available for NSW. The weighting strategy was then developed by calculating person selection probabilities, selection weights, applying a constant composite factor to the dual-phone users sample weights, and benchmarking to the latest NSW population by age group, sex and stratum.

**Results:**

Data from the NSWPHS for the first quarter of 2012 was used to test the weighting strategy. This consisted of data on 3395 respondents with 2171 (64%) from the landline frame and 1224 (36%) from the mobile frame. However, in order to calculate the weights, data needed to be available for all core weighting variables and so 3378 respondents, 2933 adults and 445 children, had sufficient data to be included. Average person weights were 3.3 times higher for the mobile-only respondents, 1.3 times higher for the landline-only respondents and 1.7 times higher for dual-phone users in the mobile frame compared to the dual-phone users in the landline frame. The overall weight effect for the first quarter of 2012 was 1.93 and the coefficient of variation of the weights was 0.96. The weight effects for 2012 were similar to, and in many cases less than, the effects found in the corresponding quarter of the 2011 NSWPHS when only a landline based sample was used.

**Conclusions:**

The inclusion of mobile phone numbers, through an overlapping dual-frame design, improved the coverage of the survey and an appropriate weighing procedure is feasible, although it added substantially to the complexity of the weighting strategy. Access to accurate Australian, State and Territory estimates of the number of landline and mobile phone numbers and type of phone use by at least age group and sex would greatly assist in the weighting of dual-frame surveys in Australia.

## Background

Since 2002 information about the health of the New South Wales (NSW) population has been obtained using the NSW Population Health Survey (NSWPHS) [1]. This survey is a continuous sample survey of approximately 15,000 persons each year. The survey is stratified by health administration area and equal numbers are selected from each of the strata, using random digit dialing (RDD) of landline phone numbers and computer assisted telephone interviewing (CATI) with one person from the selected household being randomly selected.

Because of the potential for non-coverage bias from the growing number of mobile-only phone users in the population, estimated to be 19% in Australia in 2011 [2], mobile phone numbers were included in 2012 using an overlapping dual-frame design. Coverage bias is the product of the proportion of the population not covered and the difference in the mean of the variable of interest between the covered group and the non-covered group [3]. Evidence from the National Health Interview Survey (NHIS) in the US has shown the mobile-only phone users substantial different for the health indicators: five or more drinks in one day at least once in the past year (17.5% v 30.5% - 74% higher), current smokers (14.5% v 24.3% - 68% higher), and ever diagnosed with diabetes (10.8% v 6.2% - 43% lower) [4].

The landline phone sample procedures were the same as in previous years. The mobile phone sample procedures were as follows; NSW residents were selected using RDD of mobile phone numbers using CATI and the mobile phone owner was selected. If the respondent had one or more children one child was also selected at random in order to ensure that children of people who did not have a landline were also included. Further details about the methodology, call outcomes and representation of the sample in the first quarter of 2012 are provided in Barr et al. [5], and the questions in the questionnaire are available from the survey website [1]. In the overlapping dual-frame design there are three types of phone use; mobile-only, landline-only and dual-phone users***-***people with a mobile phone and living in a household with a landline phone***—***who could now be selected though either the landline or mobile phone number sampling frames.

In the previous landline based samples for the NSWPHS, equal sample sizes were used in each stratum, even though the populations differed substantially and therefore the probability of selection varied by stratum. Moreover, as one person was randomly selected from each selected household, the probability of selection also varied by household size. Weights were calculated for use in survey estimation to account for the differences in probabilities of selection and then benchmarked to the latest NSW population by age group, sex and stratum as shown in Steel [6] and summarised in Appendix A. The use of equal probabilities to select landline phones in each stratum meant that the factor $\frac{T_{h}}{t_{h}}$, which is the ratio of phone numbers *T*_*h*_ in stratum *h* to the number of phone numbers in the sample *t*_*h*_, cancelled in the previous calculation of the weights, and so the actual number of landline phone numbers in each of the strata did not need to be known. However, with the inclusion of the mobile phone frame this is not the case and the number of landlines and mobile phone numbers in the population for each stratum needed to be estimated. In 2011 the Australian Communication and Media Authority (ACMA) estimated that there were 29.28 million mobile phone numbers and 10.54 million landline phone numbers in Australia [2]. Estimates, however, are not routinely provided by State, let alone by health administration area.

As the previous NSWPHS samples came from a single frame the weighting did not need to account for the differing chances of selection by type of phone use. However, with the inclusion of the mobile phone numbers, using an overlapping dual-frame design, dual-phone users now have an increased chance of selection because they could be selected from either frame. There is currently a growing body of knowledge on issues and methods to deal with overlapping frames as summarised in the American Association for Public Opinion Research (AAPOR): Cell Phone Task Force Report [7], and in particular the use of composite weights to adjust for the increased chance of selection of dual-phone users. However the most recent detailed description of dual frame weighting available in Australia from the Dual-frame Omnibus Survey conducted in 2012 did not need to deal with disproportionate stratification of the landline frame, data needing to be collected about children as well as adults, and how to apply an overlap adjustment [8]*.*

Hartley 1962 and 1974 [9,10] first described the calculation of these composite weights in overlapping frames. We use the notation of *A* for landline frame, *B* for the mobile frame, *Y* for the population total of interest, *y* for the estimator, *a* for landline only component, *b* for mobile only component and *ab* for dual phone users component. In this case the composite estimator is defined as *y*_*comp*_ = *y*_*a*_ + *y*_*b*_ + *y*_*λ*_ where the estimate for the overlap population is $y_{\lambda }=y_{\mathrm{ab}}^{A}+\left(1-\lambda \right)y_{\mathrm{ab}}^{B}$ with $y_{\mathrm{ab}}^{A}$ and $y_{\mathrm{ab}}^{B}$ being the estimators for persons with both mobile and landlines from frame A and B respectively and the composite factor being between 0 and 1 (0 < λ <1). Most overlapping dual frame surveys conducted to date have used a constant composite factor λ and the most common value is 0.5 [11-13]. So with overlapping dual-frames design surveys being relatively new in Australia [5,8,14,15] the use of *λ* = 0.5 as the compositing factor was considered appropriate.

Calculation of weights, in an overlapping dual-frame design, ideally requires type of phone use benchmarks as well as population benchmarks [7]. In the USA type of phone use benchmarks, at the national level, are collected using the NHIS [16], where questions on residential phone use have been included since 1963 and mobile phone use since 2003.

Currently there is no equivalent source of information on type of phone use in Australia, although landline phone use from the Australian Health Survey (AHS) conducted by the Australian Bureau of Statistics (ABS), are expected to be available in 2014 [17]. However, landline and mobile phone use questions have been included in the Roy Morgan Single Source Survey (RMSSS) since 2005 [18] for ACMA communication reporting. It was estimated in the 2010–11 report that as at June 2011, 74% of adults in Australia lived in a household with a landline and a mobile phone, 5% lived in a household with a landline but no mobile phone, and 19% lived in a household with only a mobile phone; with the highest mobile-only phone rates being in young adults (37% in 18 to 24 year olds) [2].

Because weights are used to eliminate bias that would arise from ignoring the differences in selection probabilities and also to improve estimates by adjusting to known population benchmarks, when a design change occurs it is also important to assess how the design effect changes due to weighting, using weighting effects. The design effect is the factor by which the sampling variances are larger (or smaller) than those associated with a simple random sample and no weighting [3].

This paper describes and details the final weighting strategy adopted to properly combine the data from the two overlapping sample frames in the NSWPHS and the benchmark populations used, based on the limited information available in Australia. We then compare the weight effects for the overlapping dual-frame sampling design to the previous landline frame sampling design.

## Methods

Within a stratum the landline sample was selected using equal probability of selection of landline phone numbers and then random selection of one person from the selected household. In the mobile phone sample an equal probability sample of mobile phone numbers in Australia was selected and screened for adult residents in NSW. If the respondent has one or more children one child was selected at random.

### Final weighting strategy

For the sampling design used person selection probabilities for the landline frame and mobile frame were derived as follows:

• person *ijh* from the landline frame $\pi_{\mathrm{ijh}}^{A}=\frac{t_{h}^{A}}{T_{h}^{A}}\frac{T_{\mathrm{jh}}^{A}}{N_{\mathrm{jh}}}$

• adult *i* from the mobile frame $\pi_{i}^{B}=\frac{t^{B}}{T^{B}}\frac{T_{i}^{B}}{N_{i}}$

• child *c* from parent *p* from the mobile frame $\pi_{\mathrm{cj}}^{B}=\pi_{p}^{B}\frac{N_{\mathrm{cp}}}{N_{\mathrm{cj}}}$

Where: *i* denotes an eligible person; *c* denotes a child of an eligible person; *p* denotes a parent; *h* denotes the stratum; *j* denotes a household; *N* denotes population size; *T* denotes number of phone numbers in the population; *t* denotes number of phone numbers in the sample; *A* denotes landline frame; *B* denotes mobile frame. For the design used *N*_*i*_ = 1 and *N*_*cp*_ is the number of parents that a child selected through a parent in the mobile phone frame has and *N*_*cj*_ is the number of children in the household of the parent. The weights were then the inverse *w* = *π*^− 1^ in each situation.

The sample weights of the dual phone-users were then adjusted using the composite factor λ set at 0.5. So for those dual phone-users selected from:

• the landline frame the composite weights were $w_{\mathrm{ijh}}^{\lambda }=\lambda w_{\mathrm{ijh}}^{A}$

• the mobile frame the composite weights were $w_{i}^{\lambda }=\left(1-\lambda \right)w_{i}^{B}$

Benchmarking to the reference population was then performed, as per previous years, by adjusting the weights obtained from the combined landline and mobile phone sample, by age and sex to the ABS mid-year population estimates for each stratum, *N*_*dh*_[19]. This was achieved by summing the weights for the age and sex cell *d* in stratum *h*, to produce a survey estimate of the population in that cell, ${\hat{N}}_{\mathrm{dh}}$ and then multiplying the weights by $\frac{N_{\mathrm{dh}}}{{\hat{N}}_{\mathrm{dh}}}$.

### Estimation of number of phone numbers in NSW by frame

The weights described above require the number of landline telephones in stratum *h*, $T_{h}^{A}$, and the number of mobile phone numbers in NSW, $T_{\mathrm{NSW}}^{B}$. As there was no specific NSW residential landline phone data $T_{h}^{A}$ available we divided the number of residential landline phone numbers in Australia, using the ACMA estimate [2], by the proportion of the population in that stratum, using the ABS estimates [19], after having first adjusted it by the percentage of the population who had landline phones in that stratum, using the RMSSS estimates [18]. As there was no specific NSW mobile phone data $T_{\mathrm{NSW}}^{B}$ available we divided the number of mobile phone numbers in Australia, using the ACMA estimate [2], by the proportion of the population in NSW, using the ABS estimates [19], having first adjusted it by the percentage of the population in NSW who had mobile phones, using the RMSSS estimates [18].

These procedures produce estimates as follows:

$$\begin{array}{l} T_{h}^{A}=\frac{N_{h}{\hat{P}}_{h}^{A}}{N_{\mathrm{Aust}}P_{\mathrm{Aust}}^{A}}T_{\mathrm{Aust}}^{A}\;\mathrm{and}\\ T_{\mathrm{NSW}}^{B}=\frac{N_{\mathrm{NSW}}{\hat{P}}_{\mathrm{NSW}}^{B}}{N_{\mathrm{Aust}}P_{\mathrm{Aust}}^{B}}T_{\mathrm{Aust}}^{B} \end{array}$$

Where ${\hat{P}}_{h}^{A}$ denotes the estimated proportion of people living in a household with a landline phone in stratum *h* and ${\hat{P}}_{\mathrm{NSW}}^{B}$ is the estimated proportion of people in NSW with a mobile phone.

Table 1 shows the estimated number of phone numbers by frame for NSW. We estimated that there were 3.5 million residential landline phone numbers and 9.8 million mobile phone numbers in NSW and landline numbers in the strata ranged from 23,764 in Far West health administration area to 443,603 in Hunter New England health administration area.

**Table 1:** Number of phone numbers by frame for NSW

**Health administration area (stratum for landline frame)**	**Landline frame**		**Mobile frame**	
	**% stratum with landline**	**Estimated number of lines**	**% stratum with landline**	**Estimated number of lines**
Sydney	74.0%	254015		
South Western Sydney	79.0%	406768		
South Eastern Sydney	76.0%	381287		
Illawarra Shoalhaven	82.0%	194868		
Western Sydney	79.0%	385908		
Nepean Blue Mountains	84.0%	177441		
Northern Sydney	86.0%	431456		
Central Coast	82.0%	162390		
Hunter New England	84.0%	443603		
Northern NSW	85.0%	157109		
Mid North Coast	81.0%	106940		
Southern NSW	82.0%	97434		
Murrumbidgee (inc Albury LGA)	82.8%	153043		
Western NSW	80.0%	137306		
Far West	90.0%	23764		
TOTAL	80.8%	3,513,333	85.8%	9,385,073

## Results

Data from the NSWPHS for the first quarter of 2012 was used to test the weighting strategy. This consisted of data on 3395 respondents with 2171 (64%) from the landline frame, with 17.6% being landline-only, and 1224 (36%) from the mobile frame, with 25.8% being mobile-only.

### Core weighting variables

Data needed to be available for all core weighting variables including age, sex, stratum, number of landline phones, number of mobile phones they personally have, and eligible persons in the household. If the respondent refused to provide their age or sex the interview was terminated. For the landline frame imputation was used for number of persons in household (1 if missing and 10 if greater than 10), number of landlines phones in household (1 if 0 or missing and 5 if greater than 5), number of personal mobile phones (substitute with 0 if missing and to 5 if greater than 5). For the mobile frame imputation was used for number of children in household (1 if missing and 6 if greater than 6), number of landlines in household (substitute with 0 if missing and to 5 if greater than 5) and number of personal mobile phones (substitute with 1 if 0 or missing and to 5 if greater than 5). If values could not be imputed for missing and/or erroneous core weighting variables then the record was removed from the dataset.

Data needed to be imputed, using these rules for 29 respondents for number of landline phones in the household (10 from landline frame and 19 from the mobile frame) and 26 respondents for number of personal mobile phones (15 from the landline frame and 11 from the mobile frame). The majority of respondents (97%) recruited through the landline frame were, using postcode/suburb and/or local government area provided by the respondent during the interview, in the same stratum as initially allocated, with the majority of the mismatches being within the metropolitan health administration areas (55/72; 76%) where phone numbers are more transportable. All of the respondents recruited through the mobile frame, except for 17, could be allocated to a stratum using postcode/suburb and/or local government area provided by the respondent during the interview. This resulted in 3378 respondents, 2933 adults and 445 children, for which weights could be calculated.

### Calculation of the weights

Table 2 shows the summary statistics by frame for the sample divided by number of phone lines in the population, phone lines in the household divided by eligible persons in household, person selection probabilities, person weights, and the composite weights for dual phone-users. Average person weights were 3.3 times higher for the mobile-only respondents, 1.3 times higher for the landline-only respondents and 1.7 times higher for dual-phone users in the mobile frame compared to the dual-phone users in the landline frame.

**Table 2:** Summary of the person selection probability, composite and benchmark weight statistics for each of the frames

**Group**	**Phone type**	**Description**	**Formula**	**Sum**	**Ave**	**Median**	**Min**	**Max**
Landline Frame (n = 2171)								
Adult and children (n = 2171)	All types (n = 2171)	Interviews divided by universe of phone numbers	$\frac{t_{h}^{A}}{T_{h}^{A}}$	2.68	0.0012	0.0007	0.00017	0.0041
		Lines in household divided by eligible persons in household	$\frac{T_{\mathrm{jh}}^{A}}{N_{\mathrm{jh}}}$	1216.69	0.5699	0.50000	0.11111	3.0000
		Person selection probability $\left(\pi_{\mathrm{ijh}}^{A}\right)$	$\frac{t_{{}_{h}}^{A}}{T_{h}^{A}}\frac{T_{\mathrm{jh}}^{A}}{N_{\mathrm{jh}}}$	1.59	0.0007	0.0003	0.00003	0.0082
		Selection weight $\left(w_{\mathrm{ijh}}^{A}\right)$	$\frac{1}{\pi_{\mathrm{ijh}}^{A}}$	8939582	4113.94	2864.6	121.31	35214.76
	Landline only (n = 383)	Selection weight $\left(w_{\mathrm{ijh}}^{A}\right)$	$\frac{1}{\pi_{\mathrm{ijh}}^{A}}$	1074321	2805.02	1725.43	121.31	29345.64
	Both (n = 1788)	Selection weight $\left(w_{\mathrm{ijh}}^{A}\right)$	$\frac{1}{\pi_{\mathrm{ijh}}^{A}}$	78765261	4394.00	2911.00	169.30	35214.76
		Composite weight $\left(w_{\mathrm{ijh}}^{\lambda }\right)$ (where λ = 0.5)	$$\lambda w_{\mathrm{ijh}}^{A}$$	3932630	2197.00	1455.50	84.65	17607.38
Mobile Frame (n = 1207)								
Adults (n = 1069)	All types (n = 1069)	Interviews divided by universe of phone numbers	$\frac{t^{B}}{T_{B}}$	0.14	0.0001	0.0001	0.00013	0.0001
		Mobile phones for person divided by eligible persons (where *N*_*i*_ = 1)	$\frac{T_{i}^{B}}{N_{i}}$	1168.00	1.0947	1.00000	1.00000	5.0000
		Person selection probability $\left(\pi_{i}^{B}\right)$	$\frac{t^{B}}{T^{B}}\frac{T_{i}^{B}}{N_{i}}$	0.15	0.0001	0.00013	0.00013	0.0007
		Selection weight $\left(w_{i}^{B}\right)$	$\frac{1}{\pi_{i}^{B}}$	7819874	7328.84	7655.04	1531.01	7655.04
	Mobile only (n = 284)	Selection weight $\left(w_{i}^{B}\right)$	$\frac{1}{\pi_{i}^{B}}$	2071325	7319.17	7655.04	1913.76	7655.04
	Both (n = 785)	Selection weight $\left(w_{i^{B}}\right)$	$\frac{1}{\pi_{i}^{B}}$	5748549	7332.33	7655.04	1531.01	7655.04
		Composite weight $\left(w_{i}^{\lambda }\right)$	$\left(1-\lambda \right)w_{i}^{B}$	2874274	3666.17	3827.52	765.50	3827.52
Children (n = 138)	All types (n = 138)	Parents probability of selection	$\pi_{p}^{B}$	0.02	0.0001	0.0001	0.00013	0.0003
		Number of parents divided by eligible children in household	$\frac{N_{\mathrm{cp}}}{N_{\mathrm{cj}}}$	177.57	1.2867	1.00000	0.33333	2.0000
		Person selection probability $\left(\pi_{\mathrm{cp}}^{B}\right)$	$\pi_{p}^{B}\frac{N_{\mathrm{cp}}}{N_{\mathrm{cj}}}$	0.03	0.0002	0.0001	0.00004	0.0005
		Selection weight $\left(w_{\mathrm{cp}}^{B}\right)$	$\frac{1}{\pi_{\mathrm{cp}}^{B}}$	964534	6989.38	7655.04	1913.76	22965.11
	Mobile only (n = 26)	Selection weight $\left(w_{\mathrm{cp}}^{B}\right)$	$\frac{1}{\pi_{\mathrm{cp}}^{B}}$	158842	6109.31	3827.52	1913.76	15310.07
	Both (n = 112)	Selection weight $\left(w_{\mathrm{cp}}^{B}\right)$	$\frac{1}{\pi_{\mathrm{cp}}^{B}}$	805692	7193.68	7655.04	1913.76	22965.11
		Composite weight $\left(w_{\mathrm{cp}}^{\lambda }\right)$	$\left(1-\lambda \right)w_{\mathrm{cp}}^{B}$	402846	3596.84	3827.52	956.88	11482.55
Both frames (n = 3378)								
Adults and children (n = 3378)	All types (n = 3378)	Selection weight (composite for both users) -see note (a)	$w_{i}^{U}$	10514239	3112.56	2934.56	84.65	29345.64
		Selection weight (composite for both users) scaled back to the number of respondents	$w_{i}^{U*}$	3378	1.00000	0.8698	0.04779	10.999
		Post stratification weight (benchmarked to the population by age × sex × health admin) $\left(W_{i}^{U}\right)$	$\frac{N_{\mathrm{dh}}}{{\hat{N}}_{\mathrm{dh}}}w_{i}^{U^{*}}$	7272086	2152.78	1634.97	13.54	21807

(a) The weight $w_{i}^{U}$ is the selection weight relevant to the segment of the overall sample from which the respondent was selected. For those respondents accessible through both the landline frame and the mobile phone frame it is the composite weight.

**Figure 1:**
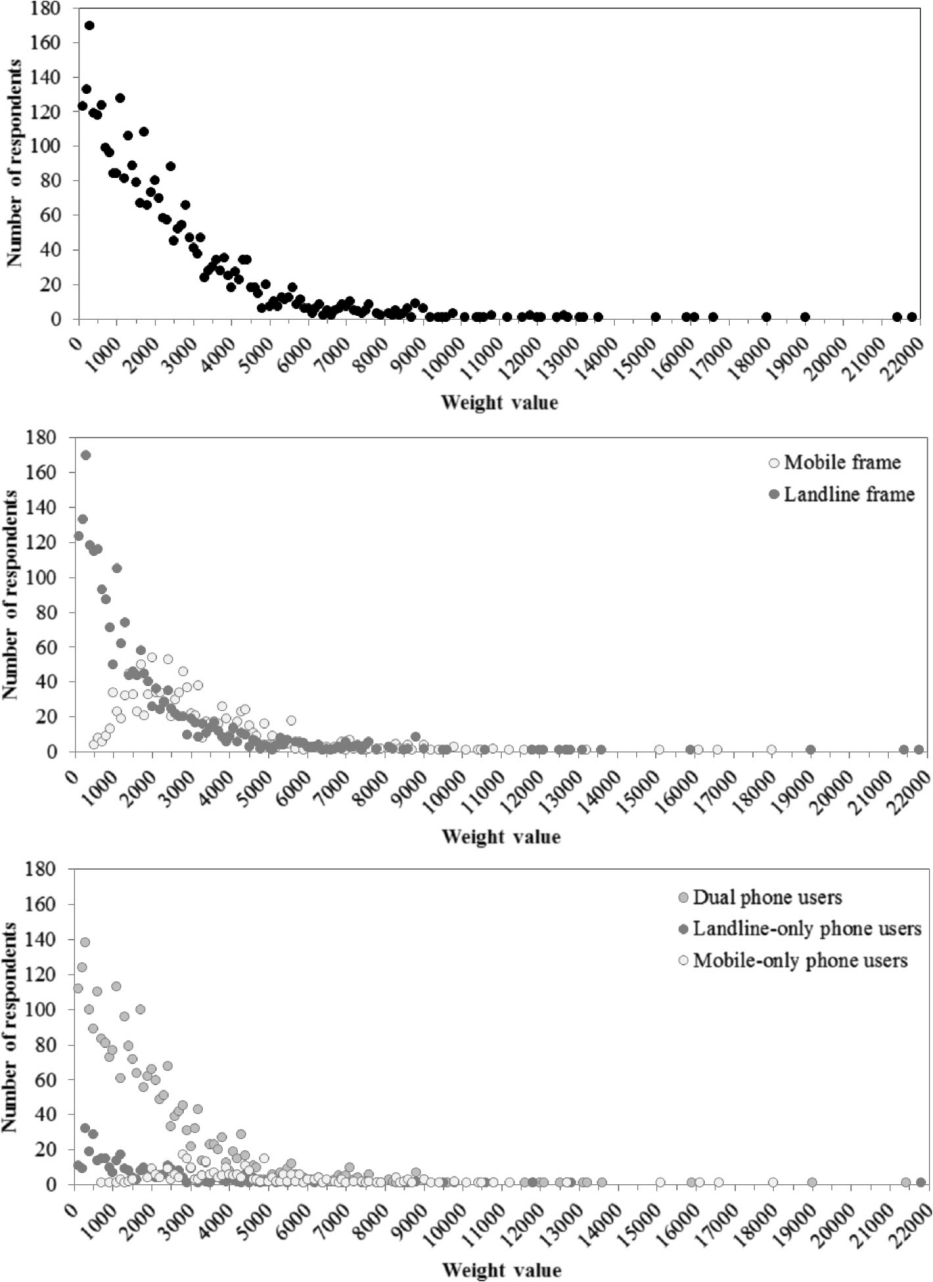
Final weights, overall, by frame and by type of phone use, quarter 1 2012 NSWPHS.

Table 2 also shows the summary statistics for the person weights, composite for dual-phone users, scaled back to the number of respondents in the sample and for the weights for the dual-frame when benchmarked to the NSW population by age group, sex and stratum. The mean final weight was 2,152, ranging from 14 for a 76 year old female dual-phone user in Far West health administration area recruited through the landline frame to 21,807 for a 76 year old male landline-only phone user in South East Sydney health administration area recruited through the landline frame. The distributions of the final weights are shown in Figure 1. Figure 1 also shows the distributions of the final weights by frame and type of phone use for comparison. Most of the variability in the weights is due to the stratification by health administration area with equal number of respondents being selected for each health administration area which is disproportionate to the populations. Because there is no geography on mobile phone numbers no stratification can occur and very few of the mobile phone frame sample comes from rural areas. Once benchmarked to the populations the urban areas get quite high weights and rural areas quite low weights. For example in Far West health administration area 82% of the weights are less than 500, whereas in South East Sydney health administration area only 2% of the weights are less than 500 and 43% are 4000 or greater.

The weight effects were calculated using $\mathrm{weff}=n\frac{\sum w_{i}^{2}}{(\sum w_{i}){}^{2}}$ where: *n* denotes sample size and *w* denotes weights [20-22]. The weight effect is the design effect due to weighting and is equal to $1+C_{W}^{2}$, where *C*_*W*_ is the coefficient of variation of the weights (i.e. the standard deviation of the weights divide by the mean of the weights) and is a standardised measure of the variation of the weights.

Table 3 shows the weight effects and coefficient of variation of the weights for each of the weighting parameters for the first quarters of 2012. As shown in Table 3 the overall weight effect for the first quarter of 2012 was 1.93 and the coefficient of variation of the weights was 0.96. Weight effects varied by: age group, from 1.55 in 25–34 years to 2.24 in 65 plus years; sex, from 1.83 in males to 1.97 in females; and stratum, from 1.41 in North Sydney health administration area, to 3.24 in Mid North Coast health administration area. The highest coefficient of variation of the weights was 1.5 for Mid North Coast health administration area. In both the previous landline only survey and the dual frame approach weights vary because of use of difference selection probabilities between strata, the sampling of one person per household and the calibration to age-sex benchmarks. Also as shown in Table 3 the weight effects for 2012 were similar to, and in many cases less than, the effects found in the corresponding quarter of the 2011 NSWPHS when only a landline based sample was used.

**Table 3:** Weight effects by weighting parameters for quarter 1 of the 2012 and 2011 NSWPHS

**Category**		**2012**						**2011**
		**n**	**SUM(WGT)2**	**(SUMWGT)**	**(SUMWGT)2**	***weff***	***C***_***w***_	***weff *** **(n = 3377)**
Age Group	0-13 years	368	7297166859	1244521	1548832668784	1.73	0.86	1.58
	14-24 years	317	5728404905	1066508	1137439271404	1.60	0.77	1.71
	25-34 years	397	4372748462	1057202	1117675032746	1.55	0.74	1.73
	35-44 years	346	4278905532	974108	948886376182	1.56	0.75	1.76
	45-54 years	489	3262991785	995006	990036601734	1.61	0.78	1.91
	55-64 years	624	2097445465	852381	726553045256	1.80	0.90	1.93
	65 plus	837	3136171943	1082361	1171505485852	2.24	1.11	1.63
Sex	Males	1429	16560322718	3600556	12964003293103	1.83	0.91	2.13
	Females	1949	13613512232	3671530	13480134523526	1.97	0.98	2.54
Health admin area	Syd	303	1698048663	585360	342646633987	1.50	0.71	1.80
	SWS	314	4303110764	892880	797234926549	1.69	0.83	1.62
	SES	213	5079590457	843566	711603697584	1.52	0.72	1.81
	IS	173	1303216701	391278	153098535888	1.47	0.69	1.82
	WS	286	3618759102	846389	716374051549	1.44	0.67	1.65
	NBM	200	1062941408	347524	120772881923	1.76	0.87	1.86
	NS	303	3343021760	846173	716008052067	1.41	0.64	1.80
	CC	210	1022421509	320135	102486405420	2.09	1.05	2.16
	HNE	314	4347558425	885170	783525875790	1.74	0.86	1.74
	NNSW	140	1082404196	300456	90273555553	1.68	0.82	1.68
	MNC	336	451722818	216328	46797881462	3.24	1.50	1.93
	SNSW	240	462055826	205377	42179613548	2.63	1.28	2.31
	M	129	885322373	241598	58369453477	1.84	0.91	1.89
	WNSW	120	1025192088	268286	71977640717	1.71	0.84	2.29
	FW	97	18833284	30750	945569265	1.93	0.97	1.80
Overall		3378	30173834950	7272086	52883238281997	1.93	0.96	2.37

NOTES: *weff* = weight effect; *C*_*w*_ = coefficient of variation of the weights.

## Discussion

The development of the weighting strategy, weighted for the person selection probabilities by frame, composite weights applied to dual-phone users, and benchmarked to the NSW population, was more complex than it had been for the previous landline frame. It was however encouraging that the weight effects were similar to those found in the previous year, when only a landline based sample was used.

The need to estimate the number of phone numbers for NSW and by stratum from the Australia figures, used to calculate the differing probabilities of selection, highlighted the desirability to be able to access accurate information at least at the State and Territory level. This is reiterated in the AAOPR report [7] which has the following comment: “A particularly troublesome issue here is that there is a dearth of highly accurate population parameters to use in weighting cell phone samples of regional, state and local areas”.

Although the first estimates of landline phone use from the AHS conducted by the ABS are expected to be available in 2014 [13], there are currently no plans to collect mobile phone use in this national survey and so the landline phone use data will be of limited use as the majority of phone users in Australia are dual-phone users [2,5,8,14,15].

Access to more accurate type of phone use benchmarks would have also allowed weighting by type of phone use. We considered using the type of phone use totals collected by RMSSS [18] to generate benchmark populations by age group, sex, stratum and type of phone use. However, after conducting a sensitivity analysis we concluded that potential errors in the type of phone use estimates provided by age group, sex and stratum, which were well below the design level of the survey, were likely to impact on the NSWPHS health indicator estimates.

The compositing factor λ used for the composite weights was set at 0.5. However the use of 0.5 as the composite factor assumes that all sampled units respond. Skinner (1991) and Skinner and Rao (1996) have explored ways to reduce non-response bias by raking the estimates to type of phone use totals from an independent source [23,24]. However, when Brick (2006) applied these to the Current Population Survey (CPS) he found that none of the suggested estimation schemes substantially reduced the non-response bias of the estimate [25]. It is possible to determine a value of this factor that minimises the sampling variance of the estimator, but this value will be variable specific. The AAOPR Cell Phone Task Force Report [7], acknowledges that variance estimation for dual frame sample designs is somewhat more complex than for single frame designs. This issue is considered by Lohr and Rao (2000) and summarised in Lohr (2009) [26,27].

Moreover, it is likely that for various reasons, the estimates obtained for the overlapping component of the population, obtained from the two sampling frames do not have the same expectation, and using *λ* = 0.5 ensures that the two frames are given equal prominence in the estimation. Although further research needs to be undertaken to explore other estimation schemes using Australian data.

## Conclusions

The inclusion of the mobile phone numbers through an overlapping dual-frame design, improved the coverage of the survey and an appropriate weighing procedure is feasible, although it added substantially to the complexity of the weighting strategy. Access to accurate Australian, State and Territory estimates of the number of landline and mobile phone numbers and type of phone use by at least age group and sex would greatly assist in the weighting of dual-frame surveys in Australia.

## Appendix A

Previous landline weighting strategy

Calculation of the raw person weight that accounts for the different selection probabilities.

The probability of selection of a household is proportional to the number of phone landline and is given by $\frac{T_{\mathrm{jh}}}{T_{h}}t_{h}$. Given a household is selected the probability a person is selected is $\frac{1}{N_{\mathrm{jh}}}$. The probability of selection of the *i* th person in the *j* th household is the product of these two probabilities and so the corresponding weight is:

$$w_{\mathrm{ijh}}=\pi_{\mathrm{ijh}}^{-1}=\frac{T_{h}}{t_{h}}\frac{N_{\mathrm{jh}}}{T_{\mathrm{jh}}}$$

Adjust the weights to agree with externally derived population benchmarks, *N*_*dh*_.

With ${\hat{N}}_{\mathrm{dh}}=\sum_{\mathrm{ijh}\in s_{\mathrm{dh}}}w_{\mathrm{ijh}}$ being the survey based estimate of *N*_*dh*_. The resulting post-stratified weight for *ijh* ∈ *d* is then

$$W_{\mathrm{ijh}}=\frac{N_{\mathrm{dh}}}{{\hat{N}}_{\mathrm{dh}}}w_{\mathrm{ijh}}$$

This allowed the factor $\frac{T_{h}}{t_{h}}$ to cancel in the calculation of *W*_*ijh*_, so that if $z_{\mathrm{jh}}=\frac{N_{\mathrm{jh}}}{T_{\mathrm{jh}}}$, then $W_{\mathrm{ijh}}=\frac{N_{\mathrm{ah}}}{\sum_{\mathrm{ijh}\in s_{\mathrm{dh}}}z_{\mathrm{jh}}}z_{\mathrm{jh}}$.

The weights are then summed to produce estimates of totals for any category and will agree with the external age-sex benchmarks. That is $\sum_{\mathrm{ijh}\in s_{\mathrm{dh}}}W_{\mathrm{ijh}}=N_{\mathrm{dh}},\;\sum_{\mathrm{ijh}\in s_{h}}W_{\mathrm{ijh}}=N_{h}$ and $\sum_{\mathrm{ijh}\in s}W_{\mathrm{ijh}}=N$

where

*i* denotes an eligible person

*h* denotes a strata *j* denotes eligible the household

*d* denotes an age-sex cell

*N* denotes population size

*n* denotes sample size

*T* denotes number of phone lines in the population

*t* denotes number of phone lines in the sample

*s* denotes the sample

## Abbreviations

AAPOR: American Association for Public Opinion Researchers; ABS: Australian Bureau of Statistics; ACMA: Australian Communication and Media Authority; AHS: Australian Health Survey; CATI: Computer Assisted Telephone Interviewing; NHIS: National Health Interview Survey; NSW: New South Wales; NSWPHS: NSW Population Health Survey; RDD: Random Digit Dialing; RMSSS: Roy Morgan Single Source Survey.

## Competing interests

The authors declare that they have no competing interests.

## Authors’ contributions

MLB developed the overall concepts and planned the study; analysis the data, wrote the methods and results, wrote the introduction and discussion and finalised the manuscript. RAF managed the data, checked the analysis programs and commented on drafts of the manuscript. PH provided development and operational advice, checked the underlying logic of the analysis and commented on drafts of the manuscript and DGS provided development and analysis advice, checked the underlying logic of the weighting and commented on drafts of the manuscript. All authors read and approved the final manuscript.

## Authors’ information

MLB is a PhD student with the National Institute for Applied Statistics Research, University of Wollongong, Wollongong Australia.

## Pre-publication history

The pre-publication history for this paper can be accessed here:

http://www.biomedcentral.com/1471-2288/14/102/prepub

## References

[B1] NSW Ministry of Health NSW Population Health Surveys http://www.health.nsw.gov.au/surveys/Pages/default.aspx

[B2] Australian Communications and Media Authority (ACMA Communications report 2010–11 2011 ACMA

[B3] Kish L Survey Sampling 1965 New York: John Wiley and Sons

[B4] Blumberg SJ Luke JV Wireless substitution: Estimates from the National Health Interview Survey. January - June 2012 2012 National Centre for Health Statistics http://www.cdc.gov/nchs/data/nhis/earlyrelease/wireless201212.PDF 21568134

[B5] Barr ML van Ritten JJ Steel DG Thackway SV Inclusion of mobile phone numbers into an ongoing population health survey in New South Wales, Australia: design, methods, call outcomes, costs and sample representativeness BMC Med Res Methodol 2012 12 177 10.1186/1471-2288-12-177 23173849 PMC3536693

[B6] Steel D New South Wales Population Health Survey: Review of the Weighting Procedure Commissioned Report to the Centre of Epidemiology and Research 2004 Sydney: NSW Department of Health of Australia

[B7] The American Association for Public Opinion Research (AAPOR) Cell Phone Task Force Report: New considerations for survey researchers when planning and conducting RDD phone surveys in the US with respondents reached via cell phone numbers 2010 Deerfield, IL: AAPOR

[B8] Pennay D Vickers N Dual-frame Omnibus Survey. Technical and methodological summary report. The Social Research Centre 2012 http://www.srcentre.com.au/docs/event-workshop-july-2012/dual-frame-omnibus-technical-report-(pennay).pdf?sfvrsn=2

[B9] Hartley HO Multiple Frame Surveys. Proceedings of the Social Statistics Section 1962 USA: American Statistical Association 203 6

[B10] Hartley HO Multiple Frame Methodology and Selected Application Sankhyā 1974 36 99 118 Ser. C, Part 3

[B11] Brick JM Cervantes IF Lee S Norman G Non-sampling errors in dual frame phone surveys Survey Methodology 2011 37 1 1 12

[B12] Lohr SL Dual frame surveys: Recent developments and challenges Proceedings of the 45^th^ Meeting of the Italian Statistical Society 2010. (Sharon Lohr, Dual Frame Surveys: Recent Developments and Challenges, David Haziza, Resampling methods for variance estimation in the presence of missing survey data, Emilia Rocco, Using auxiliary information and non parametric methods in weighting adjustments)

[B13] Wolter KM Smith P Blumberg SJ Statistical foundations of cell-phone surveys Survey Methodology 2010 36 2 203 215

[B14] Pennay D Profiling the ‘mobile phone only’ population: Results from a dual- frame telephone survey using a landline and mobile phone sample frame, ASCPRI Social Science Methodology conference proceedings ASCPRI 2010 2010 2010

[B15] Livingston M Dietze P Ferris J Pennay D Hayes L Lenton S Surveying alcohol and other drug use through telephone sampling: a comparison of landline and mobile phone samples BMC Med Res Methodol 2013 13 41 10.1186/1471-2288-13-41 23497161 PMC3607960

[B16] National Health Interview Survey http://www.cdc.gov/nchs/nhis.htm

[B17] Australian Bureau of Statistics Australian Health Survey 2011–2013 (AHS) http://www.abs.gov.au/australianhealthsurvey

[B18] Roy Morgan Single Source Survey http://www.roymorgan.com/products/single-source/

[B19] Australian Bureau of Statistics Census quickstats 2011 New South Wales: ABS http://www.censusdata.abs.gov.au/census_services/getproduct/census/2011/quickstat/1

[B20] Potter FJ A study of procedures to identify and trim extreme sampling weights Proceedings of the section on survey research methods 1990 1990 Alexandria, VA: American Statistical Association 225 230

[B21] Kish L Weighting for unequal Pi J Off Stat 1992 8 183 200

[B22] Kish L Methods for design effects J Off Stat 1995 11 55 77

[B23] Skinner CJ On the efficiency of raking ratio estimation for multiple frame surveys J Am Stat Assoc 1991 86 779 84 10.1080/01621459.1991.10475109

[B24] Skinner CJ Rao NK Estimation in dual frame surveys with complex designs J Am Stat Assoc 1996 91 349 56 10.1080/01621459.1996.10476695

[B25] Brick JM Dipko S Presser S Tucker C Yuan Y Nonresponse Bias in a Dual Frame Sample of Cell and Landline Numbers Public Opin Q 2006 70 5 780 793 10.1093/poq/nfl031

[B26] Lohr S Rao JNK Estimation in multiple-frame surveys J Am Stat Assoc 2000 101 1019 1030

[B27] Lohr S Pfeffermann D, Rao CR Multiple-frame Surveys Handbook of Statistics, Sample Surveys: Design Methods and Applications, vol 29A 2009 The Netherlands: Elsevier 71 88

